# Exploring the spatiotemporal changes in carbon storage under different development scenarios in Jiangsu Province, China

**DOI:** 10.7717/peerj.13411

**Published:** 2022-05-13

**Authors:** Xiaomian Zhang, Jun Wang, Chunlei Yue, Shuai Ma, Liang-Jie Wang

**Affiliations:** 1 Zhejiang Academy of Forestry, Hangzhou, China; 2 Co-Innovation Center for Sustainable Forestry in Southern China, Nanjing Forestry University, Nanjing, China

**Keywords:** Land use/cover, Carbon storage, Scenario analysis, InVEST model, Jiangsu Province

## Abstract

Carbon storage (CS) is closely linked to the global challenge of climate change. Land use/cover (LULC) change is the main factor driving changes in CS, and evaluating the impact of LULC changes on CS is important for carbon balance. Taking Jiangsu Province as an example, we used the Integrated Valuation of Ecosystem Services and Trade-offs model to analyze the spatiotemporal changes in CS during 2000–2015. Then we coupled it with the patch-generating land use simulation model to simulate and predict LULC and CS in 2050 under four different development plans. The results showed that LULC change in Jiangsu Province was manifested mainly as transformation of cropland to construction land (3,485 km^2^) and cropland to water body (470 km^2^). The high value area for CS was concentrated mainly in forest land, water body and grassland, whereas the low value area was concentrated mainly in construction land. During 2000–2015, CS decreased by 0.23 Tg, and during 2015–2050, CS was predicted to decrease by 0.16, 1.69, 0.02, and 0.10 Tg under the baseline, fast, slow and harmonious development scenarios. The conversion of a large amount of cropland to construction land was the main cause of CS loss. In all scenarios, the carbon loss was the largest in southern Jiangsu and lowest in central Jiangsu. It is necessary to balance the conflict between economic development and ecological protection during the process of urbanization. This study can provide an important reference for decision makers during the formulation of regional development models and ecological management strategies.

## Introduction

Terrestrial carbon is an important element of global carbon storage (CS) and plays a critical role in carbon dioxide-driven climate change (Ito, Nishina & Noda, 2016; Lal, 2004). Changes in land use/cover (LULC) are the main driving force for terrestrial CS dynamics (Rajbanshi & Das, 2021; Zhang et al., 2015). Over the past decades, rapid urbanization has altered the natural cover on land surfaces by converting extensive areas of natural and seminatural lands into urban lands (Jiang, Deng & Seto, 2013; Song, Pijanowski & Tayyebi, 2015; Su, Guo & Hong, 2019). Such conversion eventually causes a significant reduction in CS (Dai et al., 2021; Grimm et al., 2008; Li et al., 2020a; Wang et al., 2018b; Zhang et al., 2012; Zhu et al., 2021) and leads to severe environmental consequences and ecosystem degradation at multiple scales (Cao et al., 2015; De Carvalho & Szlafsztein, 2019; Tang et al., 2021). Moreover, the urbanization rate in China is predicted to reach 65.73% by 2030, which will place huge pressure on the urban ecosystem. Therefore, understanding urban carbon dynamics due to urban expansion is critical for evaluating long-term effects on ecosystem services (Wang et al., 2018a).

Ecosystem services are an important guarantee of human well-being and sustainable economic and societal development (Costanza et al., 2014; Seppelt et al., 2011; Tscharntke et al., 2005). Recently, the maintenance of urban ecosystem services has become crucial for strengthening urban ecological security and realizing sustainable management of urban ecosystems (Zhang et al., 2021). Because of its direct impact on climate regulation capacity, regional CS, including belowground CS, aboveground CS, dead organic matter CS, and soil organic CS, is considered to be an important indicator for assessing the quality and quantity of urban ecosystem services (Tao et al., 2015; Whitford, Ennos & Handley, 2001). Thus, a better understanding of spatiotemporal changes in regional CS is useful for decision-making when developing sustainable strategies for urban development. The assessment of CS is often conducted using models based on biogeochemical processes and carbon density data (Liang, Hashimoto & Liu, 2021; Quesada et al., 2018). Biochemical models are usually very complex, with high requirements on input data and diversified parameters in different regions, resulting in the accuracy of results not being guaranteed (Prestele et al., 2016). Other CS assessment models, such as the Rothamsted Carbon Model, can only model the change in soil organic carbon but ignore CS in the ecosystem (Farina, Coleman & Whitmore, 2013). The Integrated Valuation of Ecosystem Services and Trade-offs (InVEST) model is a direct and effective model to evaluate CS using carbon density data. The InVEST model is easy to use, easy to set parameters and yields relatively accurate results, which plays an important role in evaluating the impact of LULC policy on CS (Jiang et al., 2017; Posner et al., 2016). To date, InVEST has been widely used for CS evaluation worldwide. Gao & Wang (2019) used the InVEST model to analyze the spatiotemporal changes of CS in the Yangtze River Delta, China, during 1990–2015. Liang et al. (2021) and Liang, Hashimoto & Liu (2021) evaluated changes CS under different LULC scenarios in the Loess Plateau, China, with the help of the InVEST model. Therefore, the InVEST model was selected in this study to evaluate the dynamic changes of CS. Many previous studies have focused on the evaluation of multiple ecosystem services (Ma et al., 2021; Wang et al., 2021a; Wang et al., 2021b), including their trade-offs and synergies (Chen et al., 2021; Howe et al., 2014; Nelson et al., 2009; Washbourne et al., 2020). However, spatiotemporal changes in CS at the provincial scale and future changes in CS have not been thoroughly studied, especially with regard to the development needs of different cities. Analyzing the demands of different cities could help policymakers choose an optimal urbanization model and land use pattern for improving CS (Gao & Wang, 2019; Nie et al., 2020). Scenario settings are useful tools for predicting the future impacts of LULC changes on CS and can be used to produce critical information for urban development and planning (Sun & Shi, 2020; Tang et al., 2020). However, previous studies predicting changes in LULC have had some limitations. Many researchers have failed to consider policy intervention during the process of scenario setting, leading to great differences in the results (Bai et al., 2013; Shi et al., 2021). Furthermore, previous models used to predict LULC changes, such as CLUE-S, CA-Markov models, logistic-CA, ANN-CA, and future land use simulation (FLUS) models, are ineffective in revealing the potential drivers of LULC types (Cao et al., 2015; Liu et al., 2017; Xu et al., 2021). Moreover, they cannot accurately reflect the patch evolution of different land use types, especially natural LULC types such as forest land and grassland (Meentemeyer et al., 2013; Yang et al., 2020). In contrast, the patch-generating land use simulation (PLUS) model enables better exploration of the driving factors for LULC changes and their simulation at the patch level, thereby accurately simulating nonlinear changes behind LULC types (Liang et al., 2021). Compared with other models, the PLUS model has been shown to produce higher simulation accuracy, closer to the real landscape pattern indicators, and it can also be used to predict the spatial pattern of LULC under different development scenarios (Liang et al., 2021). Some scholars have used PLUS to simulate future LULC scenarios to explore changes in ecosystem services (Shi et al., 2021; Wang et al., 2022). For example, Shi et al. (2021) assessed ecosystem services under different LULC scenarios by combining the PLUS and InVEST models. Wang et al. (2022) explored the future changes of CS under different climate scenarios by integrating the PLUS and InVEST models. Therefore, PLUS can be used to accurately predict different LULC scenarios. This study coupled the PLUS and InVEST models to effectively assess the impact of different socioeconomic development and policy intervention scenarios on CS in the future, providing effective information for decision-makers to formulate future LULC development planning and management policies.

China is going through a phase of rapid urbanization, and balancing the speed of urbanization with CS is an important issue for high-quality regional development (Gao & Wang, 2019). Understanding the spatiotemporal impacts of urbanization on CS in regional areas will contribute to revealing the association of regional CS and balancing different development needs among cities (Gao & Wang, 2019; Li et al., 2020b). Jiangsu Province is among the most important provinces in China, with one of the highest comprehensive development levels. Because of rapid economic growth and urbanization, LULC in Jiangsu is undergoing dramatic changes, and the CS capacity of the ecosystem has experienced a significant decline. Thus, the sustainable development of cities and their ability to mitigate climate change will be greatly compromised (Gao & Wang, 2019). Balancing the relationship between urban expansion and CS is therefore a primary consideration for future regional development in Jiangsu Province.

In this study, we selected Jiangsu Province as a typical region for quantitatively assessing CS in response to different needs during city development. We designed four urbanization scenarios with local city planning and land policies to explore the drivers of spatiotemporal LULC changes. The study aimed to (1) analyze changes in LULC and CS from 2000–2015; (2) predict future changes in CS under different development scenarios; and (3) propose an appropriate LULC layout that can produce win-win results, balancing urban development and CS enhancement. This study can provide suggestions to policy-makers and stakeholders for improving sustainable urbanization in their area.

## Materials & Methods

### Study area

**Figure 1:**
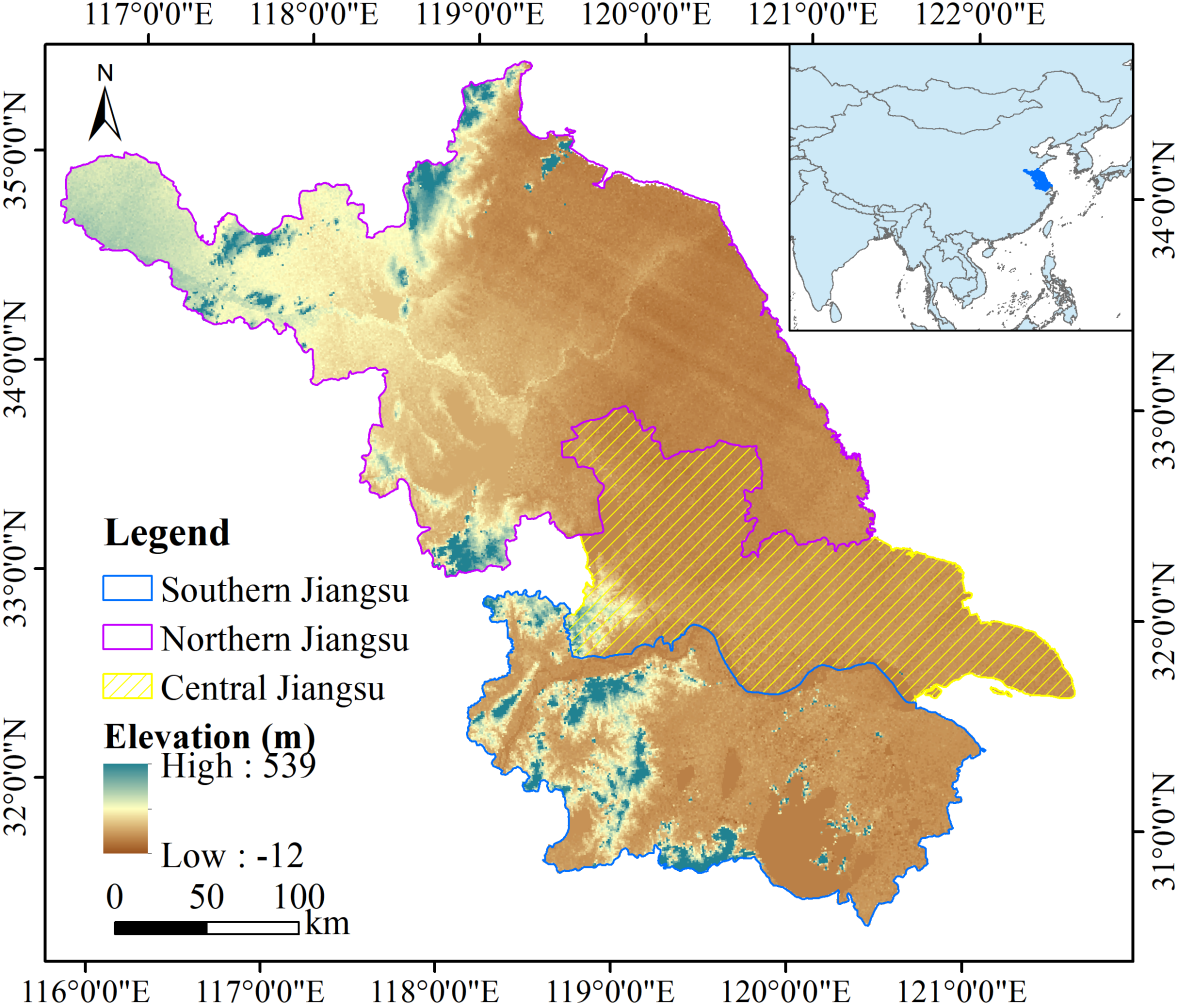
Map of the study area.

Jiangsu Province (30°45′N–35°20′N and 116°18′E–121°57′E) is located in eastern China in the lower reaches of the Yangtze River, with Shandong Province to the north, Zhejiang Province and Shanghai City to the southeast, and Anhui Province to the west (Fig. 1). This province is 10.09 × 10^4^ km^2^ in area, with 13 cities and 63 counties. The topography is mainly plains, which account for 68.8% of Jiangsu Province. The region has a subtropical monsoon climate with a mean annual temperature of approximately 15.1 °C and an average annual precipitation of 1,002 mm (Xiao et al., 2020).

 Jiangsu Province has faced considerable pressure to balance ecosystem services with rapid urbanization. In 2020, the total population of Jiangsu was 84.75 million, and its gross domestic product (GDP) was 10.27 trillion yuan. Based on the economic development status of the cities in Jiangsu, we divided the study area into three subregions: southern Jiangsu (SJ), central Jiangsu (CJ), and northern Jiangsu (NJ). Rapid population growth and gradually increasing regional inequality have made Jiangsu a more typical case study than other Chinese provinces for the assessment of changes in CS under urbanization and for the simulation of future CS changes. It provides a research test case for developing countries that face rapid urbanization.

### Data sources

LULC, precipitation, temperature, GDP, population (POP), and normalized difference vegetation index (NDVI) data with a spatial resolution of 1 km were obtained from the Data Center for Resources and Environmental Sciences of the Chinese Academy of Sciences (http://www.resdc.cn/). Data producers evaluated the accuracy of LULC based on field surveys, visual interpretations and obfuscation matrix judgments, and the results showed that the overall interpretation accuracy of LULC was over 90% (Shi et al., 2021). Six main LULC types were included: cropland, forest land, grassland, water body, construction land, and bare land. The digital elevation model (DEM) and soil data with a spatial resolution of 1 km were obtained from the National Tibetan Plateau Data Center (http://data.tpdc.ac.cn). Slope was calculated from the DEM in ArcGIS 10.3 software. Road, river, railway, and highway data were acquired from the National Earth System Science Data Center (http://www.geodata.cn/). The data used in the study are also shown in Table 1.

**Table 1:** Data used in the study.

Data	Type	Source	Resolution
Land use/land cover (LULC)	Raster	Data Center for Resources and Environmental Sciences of the Chinese Academy of Sciences https://www.resdc.cn/Datalist1.aspx?FieldTyepID=1,3	1 km × 1 km
Precipitation	Raster	Data Center for Resources and Environmental Sciences of the Chinese Academy of Sciences https://www.resdc.cn/data.aspx?DATAID=229	1 km × 1 km
Temperature	Raster	Data Center for Resources and Environmental Sciences of the Chinese Academy of Sciences https://www.resdc.cn/data.aspx?DATAID=228	1 km × 1 km
Gross domestic product (GDP)	Raster	Data Center for Resources and Environmental Sciences of the Chinese Academy of Sciences https://www.resdc.cn/data.aspx?DATAID=252	1 km × 1 km
Population (POP)	Raster	Data Center for Resources and Environmental Sciences of the Chinese Academy of Sciences https://www.resdc.cn/data.aspx?DATAID=251	1 km × 1 km
Normalized difference vegetation index (NDVI)	Raster	Data Center for Resources and Environmental Sciences of the Chinese Academy of Sciences https://www.resdc.cn/data.aspx?DATAID=257	1 km × 1 km
Digital elevation model (DEM)	Raster	National Tibetan Plateau Data Center http://data.tpdc.ac.cn/zh-hans/data/12e91073-0181-44bf-8308-c50e5bd9a734/	1 km × 1 km
Soil data	Raster	National Tibetan Plateau Data Center http://data.tpdc.ac.cn/zh-hans/data/844010ba-d359-4020-bf76-2b58806f9205/	1 km × 1 km
Road	Shapefile (polyline)	National Earth System Science Data Center http://www.geodata.cn/data/datadetails.html?dataguid=212709933867097&docId=2650	
River	Shapefile (polyline)	National Earth System Science Data Center http://www.geodata.cn/data/datadetails.html?dataguid=212709933867097&docId=2650	
Railway	Shapefile (polyline)	National Earth System Science Data Center http://www.geodata.cn/data/datadetails.html?dataguid=212709933867097&docId=2650	
Highway	Shapefile (polyline)	National Earth System Science Data Center http://www.geodata.cn/data/datadetails.html?dataguid=212709933867097&docId=2650	

### InVEST model

The Integrated Valuation of Ecosystem Services and Trade-offs (InVEST) model estimates the CS based on the aboveground biomass, underground belowground biomass, soil organic matter, and dead organic matter carbon pools. The InVEST model evaluates the CS of each pixel based on the LULC type and the corresponding carbon density. The calculation formula is as follows:

(1) \begin{eqnarray*}{C}_{\mathrm{total}}={C}_{\mathrm{above}}+{C}_{\mathrm{below}}+{C}_{\mathrm{dead}}+{C}_{\mathrm{soil}}\end{eqnarray*}

where *C*_total_, *C*_above_, *C*_below_, *C*_dead_, and *C*_soil_ are the total CS, aboveground carbon storage (AGC), belowground carbon storage (BGC), soil carbon storage (SOC), and dead carbon storage (DOC), respectively. In this study, the carbon density data for the different LULC types were obtained from the field and from some previous studies in Jiangsu Province (Chuai et al., 2014; Yu et al., 2007) (Table 2).

**Table 2:** Carbon storage (Mg/ha) of different LULC types.

LULC	AGC	BGC	SOC	DOC
Cropland	0.54	0	8.67	0
Forest land	2.65	0	11.3	0
Grassland	0.34	0	9.92	0
Water body	1.78	0	8.94	0
Construction land	0.48	0	8.1	0
Bare land	0	0	5.1	0

### LULC simulation

We simulated the spatial distribution of LULC in 2050 based on the LULC in 2015. We designed four scenarios based on different LULC policies and socioeconomic development patterns (Table 3). The baseline development (BD) scenario represents past development patterns. The slow development (SD) scenario represents moderate LULC policy and slow socioeconomic growth. The fast development (FD) scenario represents fast socioeconomic growth. The harmonious development (HD) scenario represents sustainable development.

**Table 3:** Description of different LULC policies and socioeconomic development patterns.

Development scenarios	Description
Baseline development	Moderate population growth
	Moderate GDP growth
	Moderate technological innovation
	Moderate Land policy
Slow development	Low population growth
	Low GDP growth
	Slow technological innovation
	Strict Land policy
Fast development	High population growth
	High GDP growth
	Rapid technological innovation
	Loose Land policy
Harmonious development	Low population growth
	Moderate GDP growth
	Rapid technological innovation
	Reasonable Land policy

**Figure 2:**
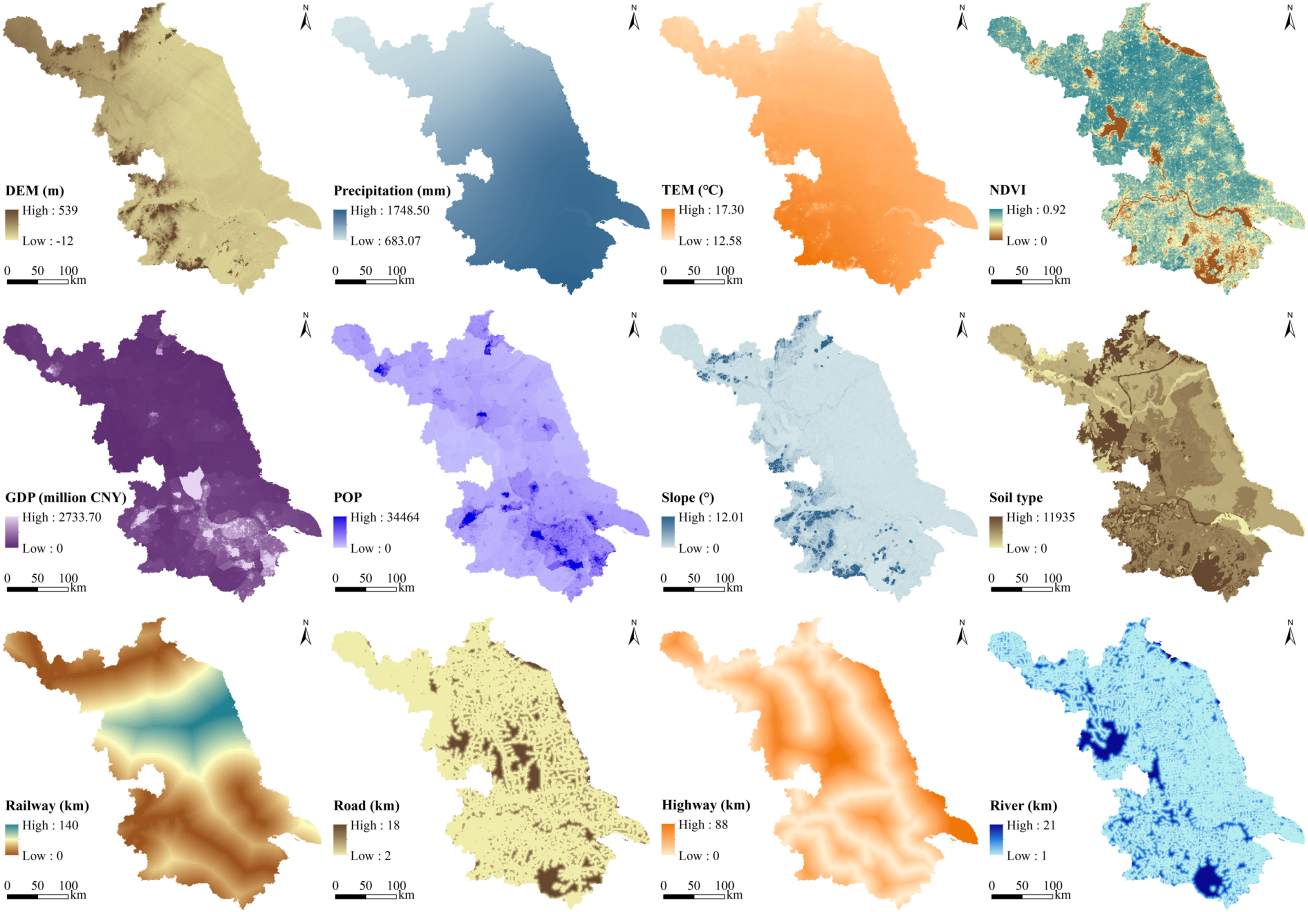
Spatial pattern of drivers of LULC change.

The spatial pattern of LULC under different scenarios was simulated with the by PLUS model. The PLUS model is an LULC change simulation model based on raster data, coupled with a new land expansion analysis strategy and a cellular automata model based on multiple types of random patch seeds (Liang et al., 2021). It can simulate the generation and evolution of multiple types of arbitrary LULC plaques, and it can also be used to explore the mechanisms of LULC change during the simulation process (Liang et al., 2021). Compared with other, similar models (CLUE-S, CA-Markov, FLUS, etc.), the PLUS model has higher accuracy (Liang et al., 2021). It can also integrate socioeconomic factors to consider LULC policy interventions. First, DEM, slope, NDVI, GDP, POP, precipitation, temperature, distance from road, distance from river, distance from railway, distance from highway, and soil type were selected as potential drivers input into the PLUS model based on previous studies (Liang et al., 2021; Guo et al., 2021) (Fig. 2). The random forest algorithm was used to mine the driving mechanism of LULC change to obtain the development probabilities for each LULC type. Then, the percentage of relative change of each LULC from 2000 to 2015 is used to represent the weight near each LULC, and the actual transfer matrix from 2000 to 2015 is used as the future LULC conversion rule (the LULC conversion rule is composed of 0 and 1, where 0 indicates that conversion is not allowed, and 1 indicates conversion is allowed). Based on the LULC in 2015, the spatial distribution of LULC in the future can be simulated according to the above settings and the input of future LULC demands. Specific parameter settings and simulation processes in the model can also be found in the reference (Liang et al., 2021).

**Figure 3:**
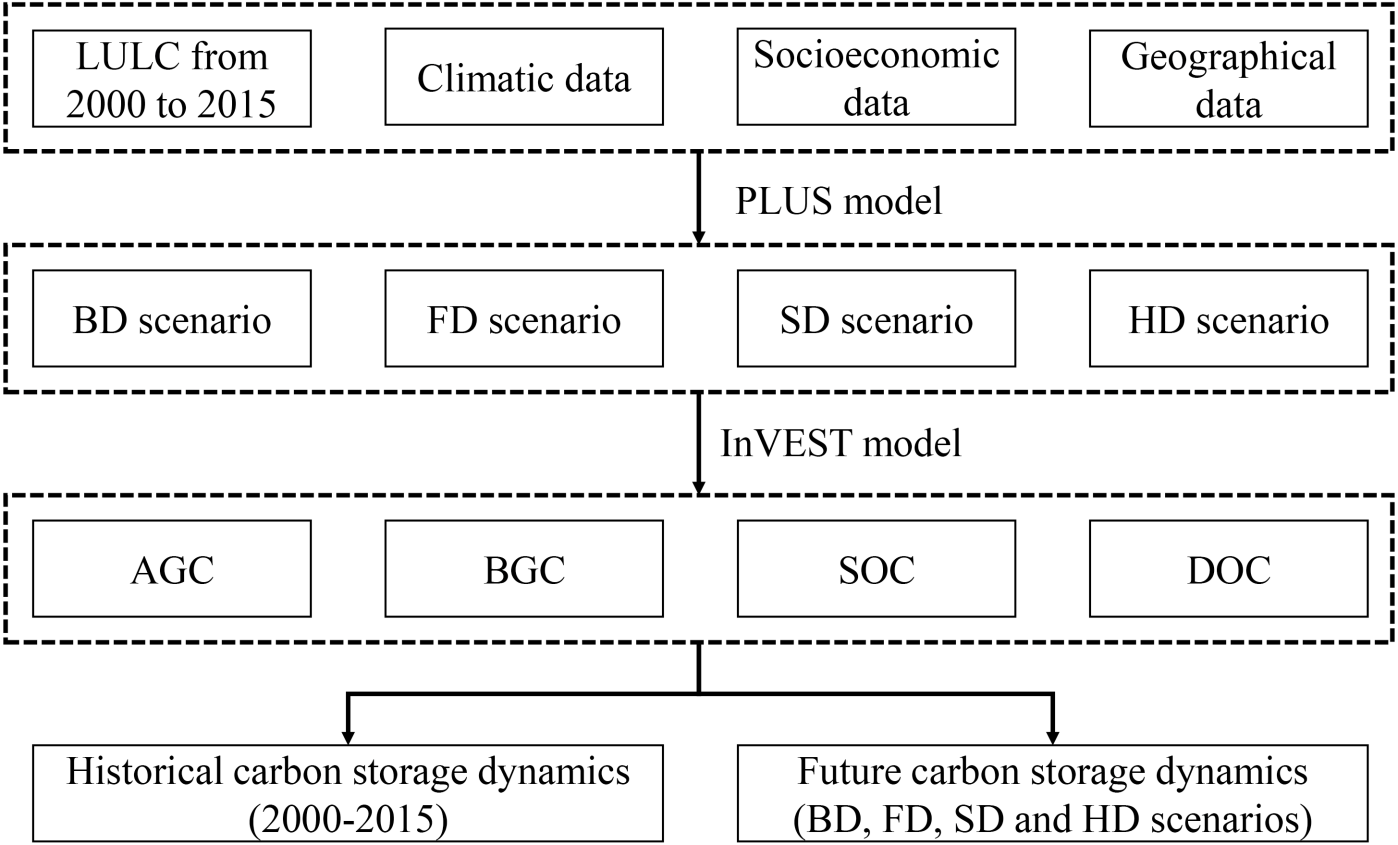
The framework for assessing carbon storage under different development scenarios.

Based on 2000 LULC, we input LULC demand in 2015, set neighborhood weight and LULC conversion rules in combination with development probability, and obtained LULC simulation results in 2015. Compared with the actual LULC in 2015, we found that the Kappa coefficient was 0.85 and the overall accuracy was 0.92. This shows that the model has high reliability. Next, using the LULC demand prediction method and prediction results shared by Liu et al. (2017) (http://www.geosimulation.cn/), the quantity of LULC types in each scenario was determined (Table 4), and the PLUS model was used to simulate the LULC spatial distribution pattern under the four scenarios based on these quantities. Finally, we examined CS under the different development scenarios. Figure 3 shows the process of embedding the PLUS model into the InVEST model.

**Table 4:** The demand for LULC types under different development scenarios (km^2^).

LULC	BD	FD	SD	HD
Cropland	65373	59864	66234	66063
Forest land	3186	1056	3281	3186
Grassland	666	433	856	746
Water body	12466	12466	12466	12466
Construction land	19209	27086	18064	18439
Bare land	16	11	15	16

**Figure 4:**
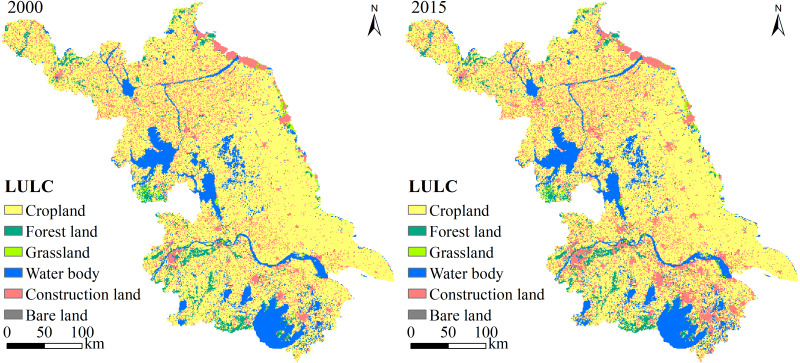
Spatiotemporal variation in LULC types in Jiangsu Province from 2000 to 2015.

## Results

### Changes in LULC from 2000 to 2015

The composition of LULC types in Jiangsu Province from 2000 to 2015 showed that cropland was the most abundant LULC type, accounting for more than 65%, followed by construction land and water body (Fig. 4). Bare land and grassland were the least abundant LULC types in Jiangsu. From 2000 to 2015, construction land and water body increased by 3,616 km^2^ and 209 km^2^, respectively, whereas cropland and forest land decreased by 3,782 km^2^ and 53 km^2^, respectively. As can be seen in Table 4, SJ had the most water body area and forest land and the least cropland compared with CJ and NJ. CJ had the smallest total area, and its areas of all LULC types were the smallest, with the exception of cropland. NJ had the most cropland, grassland, and construction land. During 2000–2015, cropland decreased by 14.28%, whereas construction land increased by 56.38%, indicating that Jiangsu Province is a rapidly urbanizing region. The construction land in CJ and NJ expanded by 33.71% and 9.28%, respectively. The LULC change was more dramatic in SJ than in NJ. From the south to the north, the urbanization process gradually slowed down, indicating that the influence of human activities gradually decreased. In addition, the reduction in forest land was greater in SJ than in NJ. Grassland and water body showed an increasing trend in SJ but showed the opposite trend in NJ. Table 5 shows that there were two types of LULC conversion in Jiangsu Province: cropland to construction land (3,485 km^2^) and cropland to water body (470 km^2^).

**Table 5:** Transformation matrix of LULC types in Jiangsu Province from 2000 to 2015 (km^2^).

LULC		2015					
		Cropland	Forest land	Grassland	Water body	Construction land	Bare land
2000	Cropland	66080	18	15	470	3485	1
	Forest land	2	3289	2	1	66	1
	Grassland	42	0	818	37	25	0
	Water body	108	0	70	11934	132	13
	Construction land	55	0	14	22	14197	1
	Bare land	0	1	0	2	0	15

### Spatiotemporal characteristics of carbon storage

**Figure 5:**
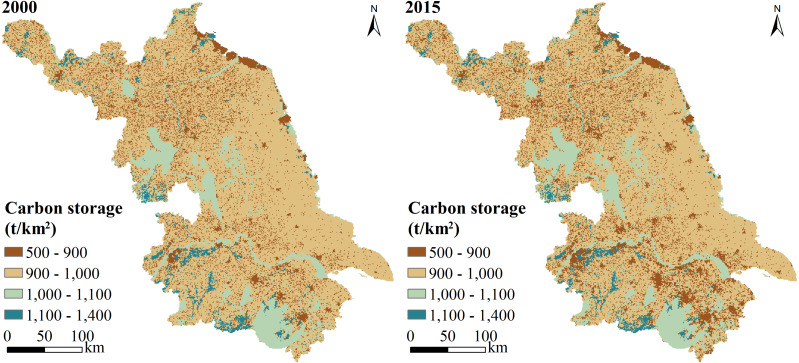
Spatiotemporal variation in carbon storage in Jiangsu Province from 2000 to 2015.

CS changed significantly between 2000 and 2015, especially in SJ (Fig. 5). The high-value areas of CS were found mainly in some large areas in the south and west-central parts of Jiangsu, although some high-value areas were located in strips in the south and north. These regions of high CS were mainly covered by forest land and water body, that is, most of SJ, the northwest of CJ, and the southwest of NJ. These regions were located in Suzhou City and south of Changzhou City, Huai’an City, and Nanjing City. The low-value areas of CS were concentrated in some regions, with a great spatial gap and a trend of expansion from the center. These areas were dominated by construction land and were widely distributed, more in SJ than in NJ. In addition, these low-value CS areas showed a clear trend of expansion over time.

The total CS in 2000 was 95.58 Tg, and it decreased by 0.23 Tg from 2000 to 2015 (Table 6). The average CS decreased from 947.09 Mg/km^2^ in 2000 to 944.84 Mg/km^2^ in 2015. The CS in SJ accounted for approximately 28% of the Jiangsu Province; it also decreased the most, from 27.10 Tg in 2000 to 26.97 Tg in 2015. Because CJ had the smallest area in Jiangsu Province, it also had the lowest CS, accounting for only 20% of the total CS and declining by 0.03 Tg from 2000 to 2015. NJ accounted for 51% of the CS in Jiangsu, and this only decreased from 48.62 Tg to 48.56 Tg between 2000 and 2015. The reduction in CS was greatest in SJ (0.13 Tg) and lowest in CJ (0.03 Tg). Regions with significant reductions in CS were located mainly in Suzhou City, the middle of Nanjing City, and Changzhou City. However, CS increased significantly in the south of Nanjing City, the south of Wuxi City, the north of Yangzhou City, the south of Suqian City, and the middle and north of Huai’an City. The reduction rate of CS in Jiangsu decreased from south to north: SJ had the highest rate, and NJ had the lowest.

**Table 6:** Carbon storage changes in Jiangsu Province from 2000 to 2015.

Region	Average carbon storage (Mg/km^2^)		Total carbon storage (Tg)	
	2000	2015	2000	2015
SJ	974.61	969.83	27.10	26.97
CJ	930.99	929.47	19.47	19.44
NJ	938.45	937.25	48.62	48.56

### Impacts of LULC change on carbon storage

LULC change is the main driving factor affecting CS. The loss of CS from 2000 to 2015 was mainly caused by a loss of SOC, which decreased by 0.22 Tg; in contrast, AGC only decreased by 0.01 Tg (Table 5). Cropland had the greatest CS (64.55 Tg), and its AGC and SOC were also the largest: 3.78 Tg and 60.75 Tg, respectively, in 2000 (Table 7). The loss of cropland caused decreases of 3.48 Tg, 0.20 Tg, and 3.28 Tg in total CS, AGC, and SOC between 2000 and 2015. The expansion of construction land and water body offset some loss of CS caused by decreases in cropland. In 2000, the CS of water body was greater than that of construction land, whereas in 2015, the increase in construction land caused its CS to exceed that of water body. The CS of construction land in 2000 was 12.26 Tg, of which 0.69 Tg and 11.57 Tg were associated with AGC and SOC, respectively. From 2000 to 2015, the AGC, SOC, and total CS of construction land increased by 0.17 Tg, 2.93 Tg and 3.10 Tg, respectively. The increase in CS was lower in water body than in construction land; it increased from 13.14 Tg in 2000 to 13.36 Tg in 2015, and AGC and SOC accounted for 16.60% and 83.40%, respectively. During 2000–2015, the CS of forest land decreased slightly from 4.69 Tg to 4.61 Tg; the AGC decreased by 0.01 Tg, and the SOC decreased by 0.06 Tg. The CS in grassland and bare land was small and showed little change.

**Table 7:** Carbon storage changes in different LULC types in Jiangsu Province from 2000 to 2015 (Tg).

LULC	2000			2015		
	AGC	SOC	CS	AGC	SOC	CS
Cropland	3.78	60.75	64.53	3.58	57.47	61.05
Forest land	0.89	3.80	4.69	0.88	3.74	4.61
Grassland	0.03	0.91	0.95	0.03	0.91	0.94
Water body	2.18	10.96	13.14	2.22	11.14	13.36
Construction land	0.69	11.57	12.26	0.86	14.50	15.36
Bare land	0.00	0.01	0.01	0.00	0.02	0.02

Changes in CS from 2000 to 2015 in Jiangsu were caused primarily by the following LULC conversions: cropland to forest land, water body, and construction land; forest land to construction land; and water body to construction land, cropland, and bare land. The conversion of cropland to construction land led to great losses in CS, and the total CS decreased by 0.22 Tg. The conversions of forest land to construction land and water body to construction land, cropland, and bare land caused CS losses of 0.04 Tg, 0.03 Tg, 0.02 Tg, and 0.01 Tg, respectively. Therefore, the loss of CS was caused mainly by urbanization. The conversion of cropland to forest land and water body was associated with CS increases of 0.01 Tg and 0.07 Tg during 2000–2015. In other words, ecological restoration projects such as returning farmland to forests and lakes increased some CS, but they did little to reverse the overall carbon loss caused by urbanization.

### Scenario setting

**Figure 6:**
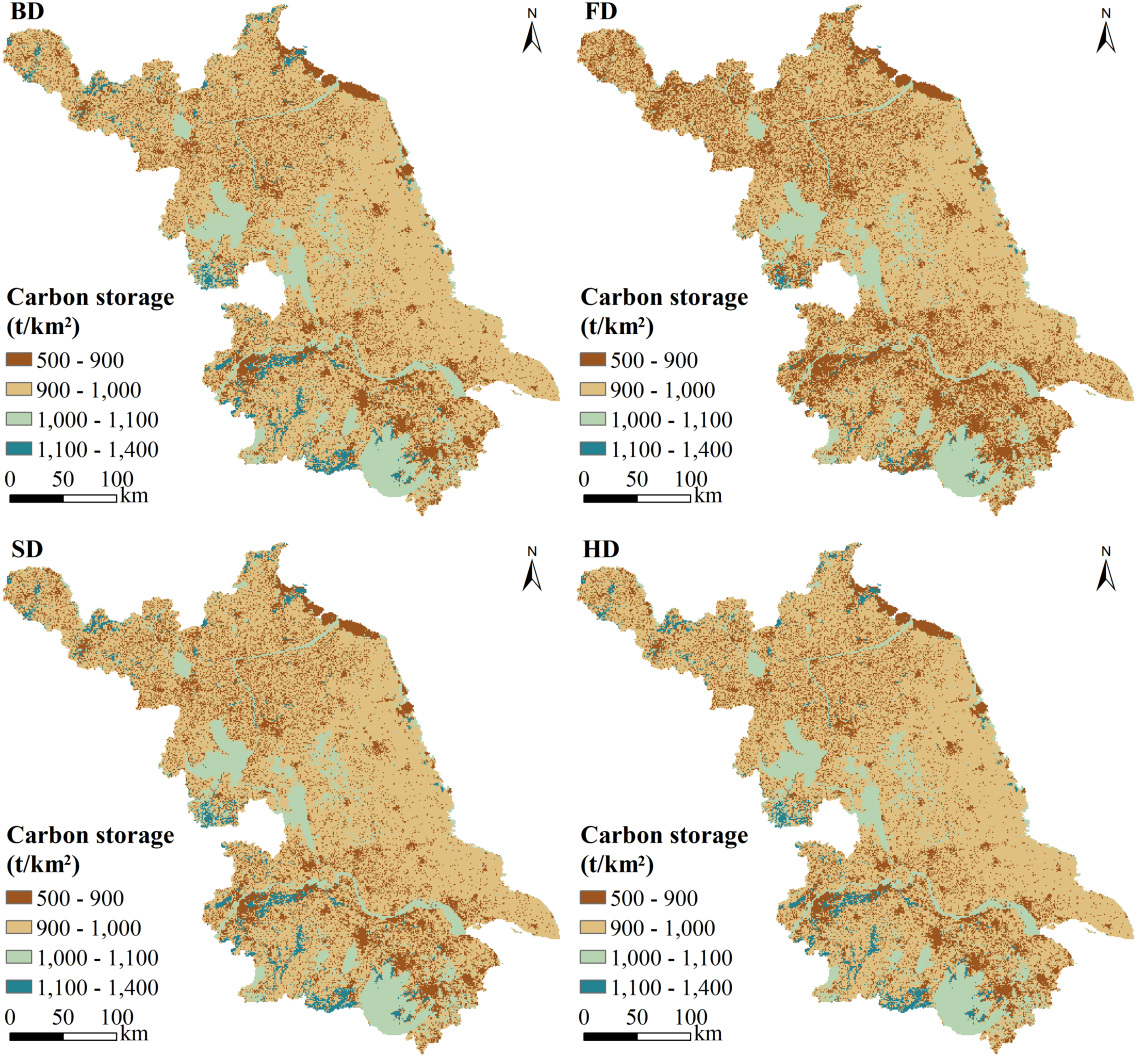
Spatiotemporal changes in carbon storage in Jiangsu Province under different development scenarios.

Figure 6 shows the predicted spatiotemporal changes in CS in Jiangsu Province under different socioeconomic development scenarios and policy interventions in 2050. Carbon losses under the BD, FD, SD, and HD scenarios were 0.16, 1.69, 0.02, and 0.10 Tg, respectively. AGC decreased by 0.03, 0.52, 0.00, and 0.02 Tg, and SOC decreased by 0.13, 1.17, 0.02, and 0.08 Tg. However, the CS of construction land increased by 1.12, 7.88, 0.14, and 0.46 Tg under the BD, FD, SD, and HD scenarios. It was clear that low-value CS regions expanded significantly under the FD scenario, primarily in the southwest and north. The carbon loss was mainly due to rapid urbanization, during which construction land expanded to occupy large amounts of cropland and forest land, mainly in the original urban periphery. In all scenarios, the greatest loss of CS occurred in SJ because of its rapid urbanization. The CS of SJ, CJ, and NJ changed to different degrees under different scenarios, but the average CS was always highest in SJ under all scenarios. The average CS was lower in NJ than in CJ under the FD scenario, but the opposite pattern was observed under the BD, SD, and HD scenarios. Under the FD scenario, the urban expansion of NJ accelerated and exceeded that of CJ. Under the other scenarios, the rate of carbon loss was still higher in the south than in the north, indicating that urbanization was more rapid in the south.

Under the FD scenario, reductions in cropland, forest land and grass land resulted in carbon losses of 5.92, 3.14, and 0.50 Tg, respectively. CS declined in all counties, among which Jiangning District, Jurong City, Yixing City, and Tongshan County had significant carbon losses. The same pattern of development occurred under the BD scenario, albeit at a slower urbanization rate than under the FD scenario. Because of the huge population pressure and rapid economic growth, the increased demand for urban infrastructure construction and urban area led to carbon loss under the FD and BD scenarios. In the BD scenario, carbon loss was still greatest from cropland (0.84 Tg), but the carbon loss from grassland (0.26 Tg) exceeded that from forest land (0.17 Tg) and became the second largest carbon loss. In both scenarios, more than half of the carbon loss came from the loss of cropland.

The distribution of CS under the SD scenario was similar to that under the BD scenario, but the carbon loss under the SD scenario was lowest. Because of slow economic and population growth in this scenario, the urbanization process was slower than that under the BD scenario. Under SD, the expansion of cropland consumed the least amount of forest land and grassland. Thus, the impact of grassland and forest land loss on CS reduction was minimal under SD relative to other scenarios. Although the reduction in grassland under SD was smaller than that under the other scenarios, the carbon loss from grassland (0.06 Tg) exceeded that from cropland (0.05 Tg) as the main source of carbon loss. Social and economic development had different influences on these scenarios: the HD scenario still maintained a pattern of forest land and grassland similar to that of SD, and reasonable plans for urban land expansion reduced the environmental pressures caused by increasing demands of social and economic development. Forest carbon loss under the HD scenario was similar to that under the BD scenario, but grassland carbon loss was lower than that under the SD scenario. In the HD scenario, although the city continued expanding, the loss of ecological land was reduced, and economic development was to some extent balanced with ecological protection.

## Discussion

### Urbanization and CS nexus relationship

Rapid urbanization and population concentration have inevitably encroached on other ecosystem types, including woodland, cropland, and grassland (Guo et al., 2021; Song, Pijanowski & Tayyebi, 2015). Therefore, there has been a significant loss of CS from urban areas. He et al. (2016) found that the loss of cropland and natural vegetation caused the loss of CS in Beijing (He et al., 2016). Jiang et al. (2017) reported that the conversion of green land ecosystems and cultivated ecosystems to construction ecosystems was the main reason for CS loss (Jiang et al., 2017). Gao & Wang (2019) found that a decrease in cropland made the largest contribution to carbon losses in the Yangtze River Delta from 1990 to 2015. Chuai et al. (2014) showed that the same patterns of LULC change could be seen in the Jiangsu coastal area. The findings in this study also indicate that reductions in cropland, forest land, and grassland around cities are the main cause of carbon stock reduction from 2010 to 2015. Surprisingly, the water body expanded greatly from 2000 to 2015, which partially offset the loss of CS. This is because since 1998, large-scale wetland restoration programs have been implemented in the middle and lower reaches of the Yangtze River, resulting in a large amount of farmland turning into water body (Guo et al., 2021; Ma et al., 2021). Moreover, we revealed the spatial variation patterns of CS in different regions of Jiangsu. This provides a theoretical basis for analysing the underlying mechanisms that control the balance between urban development and CS (Gao & Wang, 2019), which have rarely been considered in other studies.

Some studies have shown that combining CS simulation model with land use prediction model can effectively simulate potential changes in CS caused by future urban expansion (Liang, Hashimoto & Liu, 2021; Zhu et al., 2021). Here, we analyzed the spatial changes in CS during 2000–2015 and explored the intrinsic driving principles responsible for these variations. We also set up four different scenarios to predict CS patterns in 2050 based on findings from analyzing the drivers, development needs, and ecological positioning of SJ, CJ, and NJ, which are rarely considered in other studies. This scheme can help the local government and stakeholders easily evaluate CS stocks under different scenarios and provides a relatively accurate simulation of spatiotemporal results, which is critical for urban planning and the maintenance of ecosystem services (Chen, Jiang & Li, 2021; Xu, Zheng & Zheng, 2019; Yang, Huang & Liu, 2020). It is obvious that different simulation scenarios have different impacts on CS (Wang et al., 2021b). Our study showed that CS lost the most in the FD scenario and the least in the SD scenario, and the HD scenario could reduce the pressure on CS brought by economic development, which is basically consistent with the study of Gao & Wang (2019), indicating that our simulation results are credible. Under the four scenarios, CS losses showed different degrees of variation in SJ, CJ, and NJ from 2015 to 2050. Therefore, the local authorities should make development decisions that are suited to the needs of a given region (Guo et al., 2021). It is critical to take CS changes into account when planning urbanization (Li et al., 2020b). Jiang et al. (2017) analyzed the potential impact of CS changes in the Changsha-Zhuzhou-Xiangtan urban agglomeration in order to offer effective suggestions for sustainable development. Liang, Hashimoto & Liu (2021) investigated the effects of three LULC scenarios on CS services in the Loess Plateau, providing important theoretical support for local development and carbon sequestration. In future work, we should integrate multiple ecosystem services, such as water purification, flood regulation, and soil retention, into the overall management of regional development.

Because of the different developmental stages of urbanization in SJ, CJ, and NJ, it is important to propose economic development and ecological protection policies based on their individual characteristics. In SJ, with a growing population and increasing urbanization, a large amount of cropland and water body have been converted to impervious surfaces that cause significant degradation of ecosystem services. Hence, the protection of other types of ecological land, including wetlands and water body, should be enhanced by appropriate policies. In CJ, it is essential to strengthen the control of urban expansion and mitigate the enormous pressures on ecosystem services caused by population growth. At the same time, we should pay attention to the proportion of ecological land converted to impervious surfaces. In NJ, we need to raise the level of economic and social development, but at the same time, strict policies and measures should be taken to protect cultivated land and water body. Most importantly, enhancing regional ecosystem services requires comprehensive initiatives for the rational use of land resources and socioeconomic development.

## Limitations

Combining the PLUS and InVEST models is useful for simulating CS change under different scenarios. However, some limitations of the present study may benefit from further improvements. First, as urban development expands, many ecosystem services are compromised, *e.g.*, biodiversity, flood regulation, and soil retention. Hence, we should simulate multiple ESs under different scenarios and assess the temporal and spatial variations in these ESs in addition to CS. Since the latest LULC data have not been obtained, this study only analyzed the changes of CS from 2000 to 2015. In the future, yearly LULC data from 2000 to 2020 can be used to better reveal the dynamic changes in CS in the past. Additionally, the spatial resolution of the remote sensing images used in this study was 1 km, which may have impacted the simulation accuracy. We could use high-accuracy remote sensing imagery (30 m, 10 m or even 2m) to classify land-use type changes and obtain more accurate simulation results. Moreover, some important factors that may impact CS were not considered in this study; these include human cultivation habits, management methods, seasonal changes, and vegetation growth. These factors may introduce some uncertainties into the simulation results.

## Conclusions

We chose Jiangsu Province—one of China’s most urbanized provinces—as a case study to analyze changes in CS during 2000-2015 and to explore the impact of socioeconomic and policy interventions on CS under four development plans. Combining the PLUS and InVEST models is an effective means of predicting future changes in CS under different scenarios. Jiangsu Province experienced CS losses of 0.23 Tg from 2000 to 2015 and is predicted to experience further CS losses of 0.16, 1.69, 0.02, and 0.10 Tg from 2015 to 2050 under the BD, FD, SD, and HD scenarios. The CS in construction land increased, whereas that in cropland, forest land, and grassland decreased. The predicted CS losses from 2015 to 2050 were greater in SJ than in CJ and NJ; SJ had the greatest carbon loss rate and NJ had the smallest under all scenarios except for FD. Although land use conversion patterns differed among SJ, CJ, and NJ, the main driving factor causing CS losses was the transformation of cropland and ecological land into impervious surfaces. Ecological restoration projects can offset some of the CS loss caused by urbanization. Future land use strategies must balance the conflict between economic development and ecological protection. Therefore, it is necessary to optimize urban planning in different areas by considering land use patterns holistically to achieve sustainable development. The protection of ecological land should be strengthened in SJ, the control of the urban expansion rate should be strengthened in CJ, and the social and economic development should be improved in NJ. This study can help local governments contribute to green urban development and carbon emission reduction.

## Supplemental Information

10.7717/peerj.13411/supp-1 Table S1 LULC in Jiangsu Province from 2000 to 2050 (km2)

10.7717/peerj.13411/supp-2 Table S2 Carbon storage (Mg/ha) of different LULC types in Jiangsu Province

10.7717/peerj.13411/supp-3 Table S3 Transformation matrix of LULC types in Jiangsu Province from 2000 to 2015

10.7717/peerj.13411/supp-4 Supplemental Information 1 Drivers for LULC simulation Open using ArcGIS.

## Additional Information and Declarations

### Data Availability

The following information was supplied regarding data availability: The raw data are available in the Supplementary Files.
