# Supplementary figures and images for: Exploring the spatiotemporal changes in carbon storage under different development scenarios in Jiangsu Province, China
Source: PeerJ. 2022 May 13;10:e13411. doi: 10.7717/peerj.13411 (PMC9109690; doi:10.7717/peerj.13411)

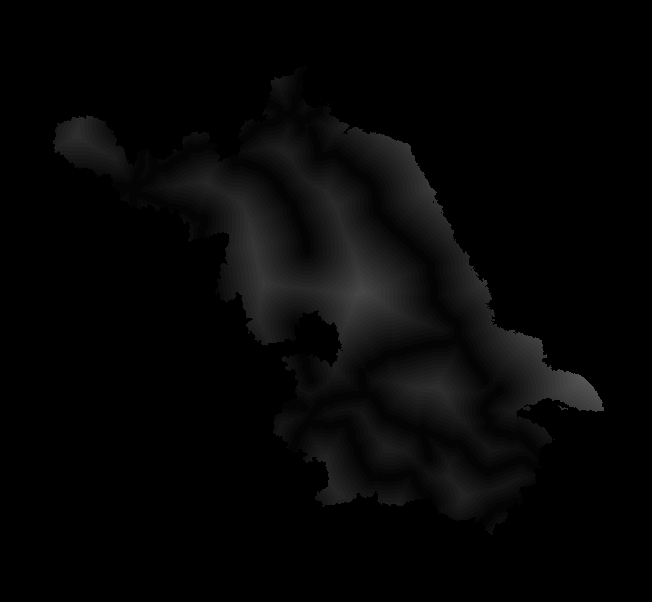

Supplement: Supplemental Information 1 — Open using ArcGIS. [file peerj-10-13411-s004.zip › driver/highway.tif]

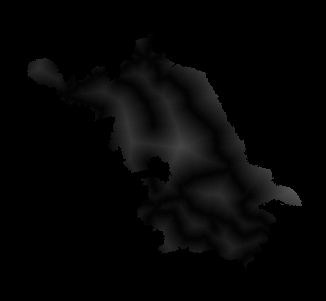

Supplement: Supplemental Information 1 — Open using ArcGIS. [file peerj-10-13411-s004.zip › driver/highway.tif.ovr]

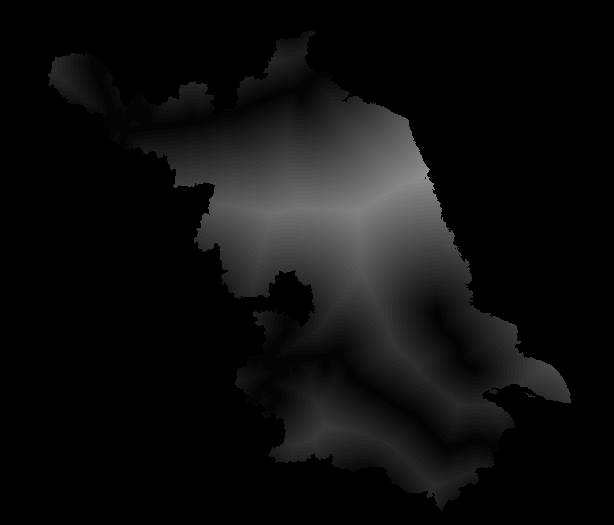

Supplement: Supplemental Information 1 — Open using ArcGIS. [file peerj-10-13411-s004.zip › driver/railway.tif]

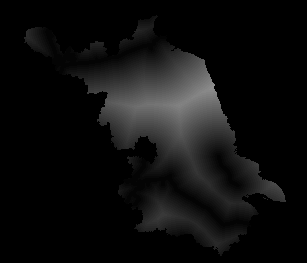

Supplement: Supplemental Information 1 — Open using ArcGIS. [file peerj-10-13411-s004.zip › driver/railway.tif.ovr]

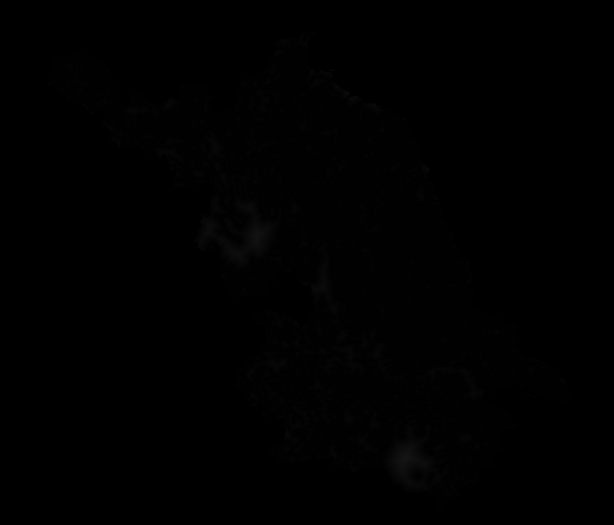

Supplement: Supplemental Information 1 — Open using ArcGIS. [file peerj-10-13411-s004.zip › driver/river.tif]

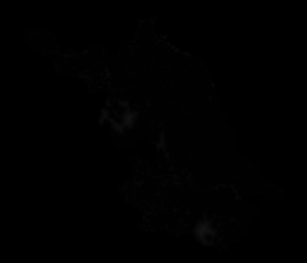

Supplement: Supplemental Information 1 — Open using ArcGIS. [file peerj-10-13411-s004.zip › driver/river.tif.ovr]

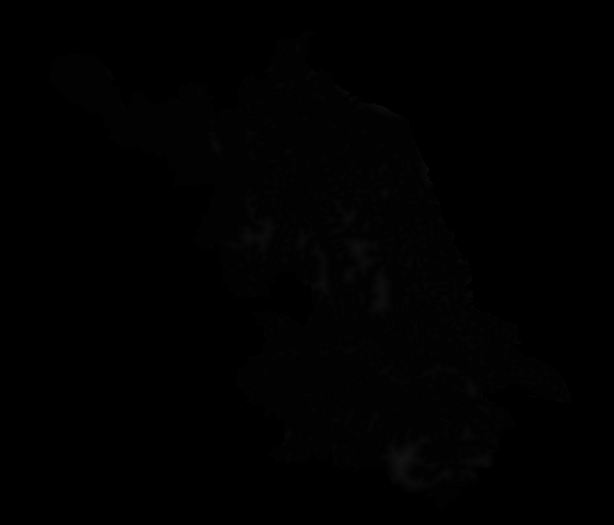

Supplement: Supplemental Information 1 — Open using ArcGIS. [file peerj-10-13411-s004.zip › driver/road.tif]

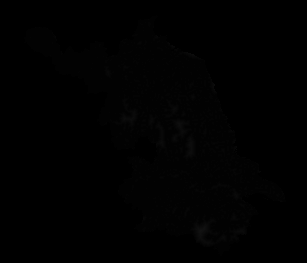

Supplement: Supplemental Information 1 — Open using ArcGIS. [file peerj-10-13411-s004.zip › driver/road.tif.ovr]
